# Somatic mutations substantially increase the per‐generation mutation rate in the conifer *Picea sitchensis*

**DOI:** 10.1002/evl3.121

**Published:** 2019-06-10

**Authors:** Vincent C. T. Hanlon, Sarah P. Otto, Sally N. Aitken

**Affiliations:** ^1^ Faculty of Forestry Department of Forest and Conservation Sciences University of British Columbia Vancouver BC V6T 1Z4 Canada; ^2^ Department of Zoology & Biodiversity Research Centre University of British Columbia Vancouver BC V6T 1Z4 Canada

**Keywords:** Base substitutions, genetic variation, mutation rate, selection within the individual, somatic mosaicism, somatic mutation

## Abstract

The rates and biological significance of somatic mutations have long been a subject of debate. Somatic mutations in plants are expected to accumulate with vegetative growth and time, yet rates of somatic mutations are unknown for conifers, which can reach exceptional sizes and ages. We investigated somatic mutation rates in the conifer Sitka spruce (*Picea sitchensis* (Bong.) Carr.) by analyzing a total of 276 Gb of nuclear DNA from the tops and bottoms of 20 old‐growth trees averaging 76 m in height. We estimate a somatic base substitution rate of 2.7 × 10^−8^ per base pair within a generation. To date, this is one of the highest estimated per‐generation rates of mutation among eukaryotes, indicating that somatic mutations contribute substantially to the total per‐generation mutation rate in conifers. Nevertheless, as the sampled trees are centuries old, the per‐year rate is low in comparison with nontree taxa. We argue that although somatic mutations raise genetic load in conifers, they generate important genetic variation and enable selection both among cell lineages within individual trees and among offspring.

**Impact Summary**

The long lifespans of trees hamper their ability to adapt to a changing environment; and yet, paradoxically, tree populations are often well adapted to local climates and evolve effective responses to changing stresses such as herbivorous insects. A plausible explanation is that trees produce many more mutations in nonreproductive cells, called somatic mutations, that can be inherited because trees do not have a segregated germline (i.e., because a tree's reproductive cells come from the same cells that build its main stem and branches). Somatic mutations could alter how trees evolve by generating more genetic variation upon which natural selection can act. We document a very high rate of somatic mutation between the base and tip of Sitka spruce trees that are centuries old.

Long‐lived trees face an evolutionary challenge: they must adapt despite generation times that are decades or centuries long. While facing substantial environmental change per generation, conifers often display strong adaptation to their local environments (e.g., in temperature tolerance and phenology; Alberto et al. 2013). Heritable somatic mutations—mutations that arise in a plant's apical meristems during vegetative growth—may contribute to adaptation by generating considerable genetic variation for selection within tree populations, as well as by enabling selection within individual trees. Such selection could act among cell lineages to purge deleterious mutations (Gaul 1964), promote the spread of beneficial mutations (Otto and Orive 1995), and slow adaptation in insect herbivores or disease‐causing fungi by confronting them with a genetic mosaic of plant defenses (Whitham and Slobodchikoff 1981; but see Folse and Roughgarden 2012). Yet the rate at which somatic mutations accumulate across the genomes of conifer trees is not currently known.

Early researchers argued that trees would have a high somatic mutation rate (see Gill et al. 1995), given the ubiquity of mutant phenotypes in horticulture and the presumption that trees undergo many mitoses per generation (from seed to seed). Strikingly, Klekowski and Godfrey (1989) observed that the incidence of mutant chlorophyll‐deficient embryos in long‐lived mangroves (*Rhizophora mangle*) is roughly 25 times higher than in short‐lived barley (*Hordeum vulgare*) and buckwheat (*Fagopyrum esculentum*). However, indirect estimates of the total mutation rate, including meiosis and the mitoses required for gamete production, are generally low for trees when calculated per year (Petit and Hampe 2006; Buschiazzo et al. 2012). Comparing neutral nucleotide substitution rates for conifers with direct estimates of mutation rates for annual rice and Arabidopsis suggests that neutral evolution is slower per year in conifers (Ossowski et al. 2010; Yang et al. 2015; de la Torre et al. 2017). This raises the possibility that somatic mutation rates may not be proportional to plant age or stature (Petit and Hampe 2006) and may contribute less genetic variation within and among individual trees than extrapolations from short‐statured plants would indicate.

Nevertheless, somatic mutations have been documented at microsatellite loci in a number of conifers (e.g., western redcedar, *Thuja plicata*; O'Connell and Ritland 2004) and during clonal growth of trembling aspen (*Populus tremuloides*; Ally et al. 2008). More recently, the first genome‐wide estimate of a somatic mutation rate for an angiosperm tree was obtained between two branch tips of a single 234‐year‐old oak (*Quercus robur*), identifying ∼40 nucleotide differences (Schmid‐Siegert et al. 2017). The inheritance of somatic mutations in progeny was later demonstrated in the same species (Plomion et al. 2018).

Somatic mutations are thought to accumulate through DNA replication errors during mitosis and through lesions that arise over time, especially in the face of environmental mutagens such as ultraviolet radiation. Large, long‐lived trees are thus expected to have high somatic mutation rates. Nonetheless, in the oak studied by Schmid‐Siegert et al. (2017), mutations accumulated at a rate that was an order of magnitude lower than predicted based on scaling up estimates from Arabidopsis according to tree size and age. This led the authors to conclude that fewer mitotic divisions occur per meter in the meristems of large statured plants and/or the structure of tree buds protects apical meristems from environmental mutagens. In conifers, however, the somatic mutation rate and the efficacy of mechanisms to protect apical meristems from mutations across the genome remain unmeasured. Gymnosperms and angiosperms are only distantly related (300–350 million years since their most recent common ancestor; Wang and Ran 2014), and they differ in both development and physical architecture, with most conifers having one dominant stem, little bifurcating branching, and a single layer of apical initials in a relatively simple meristem (Evert 2006).

We thus sought to assess the degree to which apical meristems are protected from somatic mutation by estimating the genomic base substitution rate in a long‐lived conifer. Specifically, we obtained tissue samples from 20 exceptionally tall Sitka spruce (*Picea sitchensis*), estimated to be 220 to 500 years old (Little et al. 2013), with two samples from the base and two samples from the top of each tree, separated by an average distance of 74 m (tree heights, diameters, and branch lengths in Table S1). We then used sequence capture probes to target and sequence 10.2 Mb of exons and nearby regions in each tree, out of a total estimated haploid genome size of 21 Gb (Birol et al. 2013) and transcriptome size of 182.2 Mb (Yeaman et al. 2014). We compared these sequences to identify somatic base substitutions and verified the results using Sanger sequencing.

## Methods

### SAMPLES AND PROCESSING

**Figure 1:**
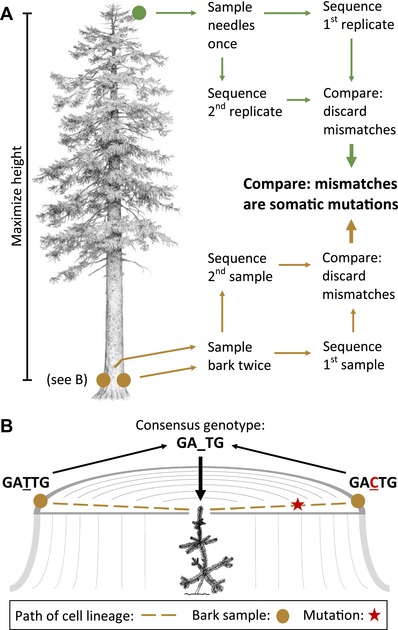
Schematic diagrams of (A) the protocol for detecting somatic mutations in 20 trees and (B) the most recent common cellular ancestor of the bark samples. To infer the genotype of the tree when it was a young seedling, we discard tree genotypes for which the genotypes of the two bark samples differ, thus eliminating nonheritable somatic mutations in the vascular cambium. Illustration in (A) by Matt Strieby.

Twenty Sitka spruce trees were chosen for their exceptional height along a one‐kilometer stretch of the Carmanah River in Carmanah Provincial Park on Vancouver Island, Canada (48.66 N, 124.69 W). We climbed each tree to obtain a sample of young foliage from a single third‐ or fourth‐order twig as high in the crown as possible, which we dried in silica beads and divided into two biological replicates (“foliage samples”; Fig. 1A). The location and size of the twigs sampled suggest they were capable of bearing cones. Each foliage sample consists of numerous leaves that were produced individually by the apical meristem (Evert 2006). We therefore expect that the consensus genotype of any pair of foliage samples is also present in the apical meristem of the sampled twig.

We also took two samples of bark from the base of each tree with a leather punch, cut a thin slice of cambium and phloem tissues from the inner surface of each sample, and dried the slices in silica beads (“bark samples”). To infer a consensus genotype of the tree as a seedling, we took the bark samples from opposite sides of the trunk. Because the most recent common ancestor of the bark samples was a cell in the center of the tree, near its meristem as a young seedling, any genotype shared by both bark samples is likely to represent the genotype of the young seedling itself (Fig. 1B). We required agreement in the genotypes inferred for the two bark samples and for the two foliage samples to reduce the frequency of false positives in our data.

Genetic differences between the bark (lower) and foliage (upper) samples thus represent somatic mutations that occurred in the apical meristem during height growth, suggesting that—in the absence of chimerism in the apical meristem—they are likely heritable. Although one might initially think that a somatic mutation rate could also be estimated by comparing the two bark samples from a tree, the radial growth pattern of trees implies that a bark sample will consist of cells that have descended according to an almost star‐like phylogeny, with a most recent common ancestor also near the center of the tree, reducing the genetic difference expected to accumulate between the two bark samples (Supporting Information Note 1).

We extracted DNA separately for all four samples from each tree and used a CTAB protocol modified from Zeng et al. (2002) for bark samples and the Nucleospin Plant II kit for foliage samples (Macherey‐Nagel, Düren, Germany). Sequence capture probes (Roche Nimblegen, Madison, WI) were designed for 4354 of the 79,745 exon‐rich contigs captured in a previous study of *Picea glauca* × *engelmannii* hybrids (Suren et al. 2016), chosen because they were captured and sequenced most successfully when tested across multiple Sitka spruce (Elleouet 2018). The total length of these target regions in each tree was 10.2 Mb, representing 0.05% of the genome and roughly 5.6% of the transcriptome (Birol et al. 2013; Yeaman et al. 2014). The prepared reduced‐representation libraries were indexed and sequenced on four lanes of an Illumina HiSeq 4000 with 100 base pair paired‐end reads at Genome Quebec (Montreal, Canada).

### SNP CALLING AND FILTERING

We aligned both on‐ and off‐target reads to the draft reference genome of a closely related natural hybrid, *Picea glauca* × *engelmannii* (PG29 version 4.1; Birol et al. 2013; Warren et al. 2015), using NextGenMap version 0.5.0 (Sedlazeck et al. 2013). Before aligning, the targeted contigs of the highly fragmented reference genome were assembled into pseudo‐scaffolds separated by 400 “N”s to facilitate the use of bioinformatic programs downstream. We marked PCR duplicates with Picard MarkDuplicates version 2.8.1, and we applied HaplotypeCaller with the flag ‐ERC GVCF and GenotypeGVCFs with the flag *‐all_sites* to create a VCF file of both invariant and variable sites (GATK version 3.7; Van der Auwera et al. 2013). In our usage, “sample genotype” means the genotype of a bark or foliage sample at a site in the genome, whereas “tree genotype” refers collectively to all four sample genotypes for a tree at a site.

Each nucleotide (site) sequenced was filtered in two steps, one filtering out sites with poor‐quality sequence data (“site‐level filters”) and the next filtering out weakly supported sample genotypes (“genotype‐level filters”; Table 1). We used two separate sets of filtering criteria at both the site and genotype levels, one identifying only higher‐confidence mutations with more conservative filters (“higher‐confidence pool”), and one identifying a larger pool of lower‐confidence mutations with less conservative filters (“lower‐confidence pool”).

**Table 1:** Quality filters for sequence data based on statistics from GATK version 3.7

		Higher‐confidence and rare	Higher‐confidence and frequent	Lower‐confidence and rare	Lower‐confidence and frequent
Site‐level filters (all sites)	Missing data per site	≤85%	≤85%	≤85%	≤85%
	Site depth	≤7000	≤6000	≤7000	≤6000
Site‐level filters (SNP‐only sites)	ReadPosRankSumTest	–1.3 to 1.6	–0.6 to 0.7	None	–0.6 to 0.7
	BaseQualityRankSumTest	–0.7 to 1.3	–0.6 to 1.0	None	None
	StrandOddsRatio	None	≤1.4	≤1.4	≤1.4
	FisherStrand	None	≤12	None	≤12
Genotype‐level filters (all sites)	Genotype depth	12–75	12–70	None	10–70
Genotype‐level filters (SNP‐only sites)	Heterozygotes: reads supporting one allele	23–77%	40–60%	20–80%	35–65%
	Homozygotes: reads supporting one allele	≥97%	≥98%	≥95%	≥98%
	Genotype quality (PL)	≥15	≥53	>0	>0

*Note*. Tree genotypes at sites meeting these criteria were retained and checked for mutations. A site was said to have missing data if there was no genotype call for ≥85% of the 80 samples.

This custom filtering approach addresses the high frequency of alignment errors generated by the incomplete and fragmented reference genome. As a proxy for the expected number of false positive candidate mutations, we counted the number of tree genotypes across all sites for which the same sample genotype was inferred for one of the bark and one of the foliage samples, with a different sample genotype inferred for the other bark and foliage samples. We calibrated the filters for the higher‐confidence pool by iteratively increasing the number and stringency of filters until this proxy for false positives was small relative to the number of candidate mutations.

We applied the site‐level filters first. Because we found that genotype calls at sites with a high minor allele frequency (MAF) across all trees were less reliable—presumably because they indicate alignment errors—we divided variable sites into two categories, rare (MAF ≤ 0.05) and frequent (MAF > 0.05), applying more stringent filters on sites where the minor allele was frequent. We first discarded indels and multiallelic sites and then applied a series of filters that do not alter the relative proportions of mutant and nonmutant sites: the site‐level filters labeled “all sites” in Table 1. Because the StrandOddsRatio filter is not applied to rare, higher‐confidence sites, the higher‐confidence pool is not a perfect subset of the lower‐confidence pool. We then separated invariant and variable sites and refined the latter using the site‐level filters labeled “SNP‐only sites” in Table 1 (GATK version 3.7; Van der Auwera et al. 2013).

For the genotype‐level filters, we defined homozygous‐reference, heterozygous, and homozygous‐alternate genotypes (relative to the hybrid spruce reference) by setting criteria for the proportion of reads supporting the reference and alternate alleles. We also applied the genotype‐level filters listed in Table 1 to all 80 samples independently (GATK version 3.7; Van der Auwera et al. 2013). These filters are biased against calling heterozygous genotypes, reducing the number of observed mutations and affecting rare and frequent sites differently, a bias that we corrected statistically (see below).

Only the filters labeled “all sites” in Table 1 were applied to invariant sites as well as variable sites. We used these filters to produce a broad set of good‐quality invariant and variable sites that are the basis for the calculation of the search space (see *I* and *V*, below).

### CANDIDATE MUTATIONS

For sites that passed all the filters, tree genotypes were retained and checked for mutations only if all four sample genotypes passed the filters. We considered any tree genotype for which both bark (lower) samples have the same genotype, both foliage (upper) samples have the same genotype, and the bark and foliage genotypes differ to be a candidate somatic mutation. We thus obtained two sets of candidate mutations, one each from the higher‐ and lower‐confidence pools.

For candidate mutations in the lower‐confidence pool, we first used the Integrative Genomics Viewer (IGV; Robinson et al. 2011) to visually examine raw sequence alignments and identify false positives, building upon a set of criteria for true mutations from Keightley et al. (2014). These criteria are consistent with alignment errors, perhaps caused by misaligned paralogs. We considered a candidate mutation to be a false positive if one of the following was true:

- All reads that showed the candidate mutant allele also showed a unique allele at a nearby site in the heterozygous samples, and at least one of the homozygous samples contained reads supporting the two alleles.
- At the candidate site and at a nearby polymorphic site, there were three haplotypes in the heterozygous samples, each supported by multiple reads.
- All four samples contained at least one read supporting the candidate mutant allele.

Candidate mutations in the lower‐confidence pool that passed the IGV criteria were verified using Sanger sequencing of one foliage sample and at least one bark sample, as were all candidate mutations in the higher‐confidence pool. We designed primers for each mutation, amplified the fragment, and sent it to the Sequencing and Bioinformatics Consortium (the University of British Columbia, Vancouver, Canada) for sequencing. If good‐quality Sanger sequence showed no mutant allele in a putatively heterozygous sample, we considered the candidate mutation to be a false positive. If the mutant allele was both confirmed in a putative heterozygous sample and absent in a putative homozygous sample from a tree, we considered the candidate mutation to be a true positive.

For verified mutations, mutant codons were located within the longest open reading frames produced by Yeaman et al. (2014) for their *Picea glauca* × *engelmannii* transcriptome, from which the impact on the predicted protein was inferred (Table 2).

**Table 2:** Mutations and their characteristics

Contig	Position	Mutation	CpG site	C‐containing dipyrimidine site	Effect
ALWZ04S2162146.1	18479	G/C to A/T	Yes	Yes	Non‐synonymous
ALWZ04S2011522.1	5666	G/C to A/T	No	No	Not in exon
ALWZ04S1889965.1	2816	G/C to A/T	No	Yes	Synonymous
ALWZ04S2907496.1*	48235	G/C to T/A	No	No	Non‐synonymous
ALWZ04S1913287.1*	11681	A/T to T/A	No	No	Not in exon

*Note*. The positions give the number of the mutant base pair within the indicated contig in the PG29 version 4.1 reference genome. Asterisks (*) indicate mutations from the lower‐confidence pool.

### THE SEARCH SPACE AND THE MUTATION RATE

To convert the number of verified mutations into a rate, we estimated the total number of tree genotypes at which we could have detected a mutation had one occurred. In brief, we first included the tree genotypes (from variable sites) that were checked for mutations, and we then used the fraction of tree genotypes at variable sites that passed filters to determine how many tree genotypes from invariant sites to include as well. This procedure was necessary because some of the filters applied only to variable sites, including mutant sites (SNP‐only filters, Table 1). The size of the search space was calculated separately for the higher‐ and lower‐confidence pools.

We also corrected a bias in the genotype‐level filters against heterozygotes, observed as a decrease in heterozygosity, on average, after the filters were applied to variable sites. Because the mutations we verified consist of two homozygous bark samples from the bottom of the tree and two heterozygous foliage samples from the upper crown, such bias also implies that the filters retain mutant genotypes at a slightly lower rate than wild type genotypes. To compensate, we reduced the size of the search space by 7% for the higher‐confidence pool and by 1% for the lower‐confidence pool (Supporting Information Note 2).

More precisely, after excluding indels and multiallelic sites, and after discarding tree genotypes with missing data in any of the four samples, the size of the search space was calculated as

(1) (I+V)σ rare +σ freq V

where *I* is the number of tree genotypes at invariant sites that passed the all‐sites filters, *V* is the number of tree genotypes at variable sites that passed the all‐sites filters, and σ rare  and σ freq represent the number of tree genotypes at variable sites with MAF ≤ 0.05 and MAF > 0.05, respectively, that also passed the SNP‐only filters and were subsequently checked for mutations (accounting for the heterozygosity correction, applied separately to the higher‐ and lower‐confidence pools; Supporting Information Note 2). The fraction in (1) thus represents the reduction in search space due to the filters that could only be applied to variable sites. *I* and *V* depend on the choice of filters (Table 1), as these filters determine the number of tree genotypes in the search space. Because a mutation turns an invariant site into a variable site with a low MAF, we calculated *I* and *V* using the rare‐allele filters.

To calculate the mutation rates for the higher‐ and lower‐confidence pools, the number of verified mutations from a pool was divided by the size of the corresponding search space, and then divided by two to translate the mutation rate at diploid sites to a haploid rate. This mutation rate is given within a generation, rather than per generation, because the age of the trees is unlikely to be the average generation time for the species and because our estimate excludes mutations that arise during seed production or early development. We divided the mutation rate within a generation by 360 years, the midpoint of the age range for similar old growth trees in the same stand (220–500 years; Little et al. 2013), to derive the per‐year mutation rate. We also obtained per‐generation mutation rates and estimates of generation time or lifespan for several other species, for comparison, and calculated per‐year mutation rates in the same way (Table S2).

## Results

We sequenced paired tissue samples from the tops and bottoms of 20 old‐growth Sitka spruce trees to identify somatic mutations. Overall, we obtained 276 Gb of Illumina sequence data, with a mean read depth per sample of 34 over the 10.2 Mb target regions and 71% of target bases covered to a depth of at least five in all four samples from each tree. Overall 97.6% of reads could be aligned to the reference, and 23.7% of reads captured by our sequence probes were marked as PCR duplicates.

After SNP calling, we applied two filtering strategies to produce one higher‐confidence pool of variants and one lower‐confidence pool of variants. Before filtering, the sum over all variable sites of the number of tree genotypes (i.e., two bark and two foliage samples from a tree at a genomic site) with a depth of at least five in all four samples was 12,289,511. After applying all filters, there remained a total of 2,291,498 tree genotypes at variable sites in the higher‐confidence pool (18.6%) and 3,762,052 tree genotypes at variable sites in the lower‐confidence pool (30.6%). Many of these sites were in exons (Yeaman et al. 2014; 39.8% for the higher‐confidence pool and 37.5% for the lower‐confidence pool) and a roughly equal number were on the same genomic contig as an exon (higher‐confidence pool: 37.5%; lower‐confidence pool: 37.7%). Because the draft reference has 3,033,322 contigs, of which just 74,436 contain known exons, the latter can be considered near exons (Yeaman et al. 2014). Finally, about two‐thirds of these sites were in the 10.2 Mb target regions (higher‐confidence pool: 69.6%; lower‐confidence pool: 65.1%).

We then calculated the size of the search space using (1). For the higher‐confidence pool, the number of tree genotypes at invariant sites that passed the all sites filters (*I*; see Methods) was 124,959,176, and the number of tree genotypes at variable sites that passed the all sites filters (*V*) was 4,903,088 (for the lower‐confidence pool: *I* = 276,788,336 and *V* = 12,835,346). After the heterozygosity correction (Supporting Information Note 2; Table S3; Table S4), σ_rare_ was 1,596,139 and σ_freq_ was 538,079 for the higher‐confidence pool (for the lower‐confidence pool: σ rare = 2,570,888 and σ freq = 1,153,990). The cumulative search space across all trees was therefore estimated to be 56.5 Mb for the higher‐confidence pool and 84.1 Mb for the lower‐confidence pool, comprising both a subset of the target regions for each tree and also nontarget sites covered by off‐target reads.

Comparing the consensus genotypes from the bark samples to the foliage samples using the conservative filters yielded 24 candidate base substitutions in the higher‐confidence pool. Of these, Sanger sequencing verified three, identified 16 as false positives, and was inconclusive in five cases. When we applied the IGV criteria (see Methods) to the 24 candidate mutations, only the three mutations later verified by Sanger sequencing were predicted to be true positives. Of the five candidate mutations that we could not Sanger sequence, two appeared to be paralogs with three haplotypes, and three had roughly equal numbers of reads supporting each allele in all four samples from the tree, suggesting that all five were false positives.

Using more relaxed filters, we obtained 141 candidate somatic mutations in the lower‐confidence pool. For those which were not included in the higher‐confidence pool, we first used the IGV method to eliminate false positives and rejected all but three. For these three, Sanger sequencing verified two as somatic mutations but identified the third as a false positive. In total, across both the higher‐ and lower‐confidence pools, we verified five somatic base substitutions within the genomic regions scanned.

For each of the five verified somatic base substitutions, a homozygote mutated to a heterozygote containing an allele that was unique among all trees. No tree or genomic scaffold contained more than one mutation: we did not detect any mutations for 15 of the trees sampled. Three mutations were G/C to A/T transitions, of which two occurred at C‐containing dipyrimidine sites (Table 2) known to be susceptible to UV‐induced damage (Friedberg et al. 2006). Three of the five mutations aligned to exons within the *Picea* transcriptome assembly (Yeaman et al. 2014), generating two nonsynonymous and one synonymous change, whose functional consequences are unknown. The remaining two occurred outside of the hybrid spruce transcriptome, of which one mapped most closely to an untranscribed paralog of a duplicated gene in the PG29 version 4.1 reference (ALWZ04S1913287.1).

**Figure 2:**
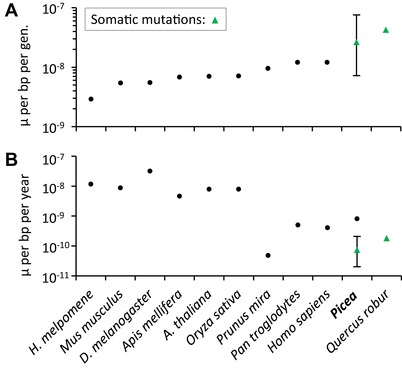
Mutation rates per generation (A) and per year (B) for multicellular species with sufficient data available. Per‐year rates were calculated using a reported age, an estimate of generation time, or an average of two estimates of generation time (raw data and references in Table S2). For the trees (*Prunus*, *Picea*, and *Quercus*), the mutation rates were estimated in exceptionally long‐lived specimens; for *Picea*, the lower per‐year mutation rate is our estimate for *P. sitchensis* and the higher per‐year mutation rate is the average neutral substitution rate for *P. glauca*, *P. sitchensis*, and *P. abies* (De La Torre et al. 2017). Error bars represent the Poisson confidence intervals.

We estimate a somatic base substitution rate of 2.7 × 10^−8^ (exact Poisson 95% confidence interval [CI] [7.2 × 10^−9^, 7.6 × 10^−8^]) per base pair within a generation for the high‐confidence pool, or 7.4 × 10^−11^ (CI [2.0 × 10^−11^, 2.1 × 10^−10^]) base substitutions per base pair per year (Fig. 2). We note, however, that these confidence intervals account only for the inherent stochasticity in observing mutations (as captured by a Poisson distribution), and they fail to account for mutation rate heterogeneity or potential biases in the methods (see Discussion). Uncertainty in the ages of the trees studied may also affect the per‐year estimate (Table S2). If we use the endpoints of the age range rather than the midpoint of 360 years (Little et al. 2013), we obtain per‐year base substitution rates of 1.2 × 10^−10^ (220 years) or 5.3 × 10^−11^ (500 years).

Extrapolating our estimated mutation rate from the number of postfiltered sites to the entire diploid genome of Sitka spruce (doubling the haploid genome size of ∼21 Gb; Birol et al. 2013), we expect 1134 base substitutions between the original seedling and the apex of the trees studied. If we assume IGV did not eliminate any true mutations and then expand the search space to include all tree genotypes that passed the relaxed filters (i.e., combining the higher‐ and lower‐confidence pools), we estimate a similar somatic base substitution rate of 3.0 × 10^−8^ (CI [1.2 × 10^−8^, 6.9 × 10^−8^]) per base pair within a generation using the full set of five verified mutations.

Some mutations likely did not pass the SNP‐only filters, and although these are false negatives in the sense that they are not among the five verified mutations, we accounted for the loss of these potential mutations when calculating the search space to obtain an unbiased estimate of the mutation rate. Under the assumption that new mutant samples and preexisting heterozygotes pass the SNP‐only filters at the same rate, we used the fraction of tree genotypes at variable sites that passed the SNP‐only filters to reduce the number of invariant sites in the search space to the same extent. The close agreement between the mutation rates estimated from the higher‐ and lower‐confidence pools supports the view that we adjusted the search space appropriately when applying filters of different stringency. Finally, the low rate of disagreement among pairs of bark sample genotypes or pairs of foliage sample genotypes (<0.001%) suggests that mutant genotypes are unlikely to have been miscalled as wild‐type during the SNP calling process (see Methods).

## Discussion

The somatic base substitution rate that we report for old‐growth Sitka spruce is among the highest per‐generation mutation rates for any eukaryote (Fig. 2A), although our estimate is within a generation and does not include mutations from error‐prone meiosis (Magni 1963). We note that the mutation rates presented here may be affected by regional mutation rate variation within the genome because the search space is a small fraction of the total genome size. In particular, our estimates may be downwardly biased if mutation rates are lower within than outside of exons (e.g., Ossowski et al. 2010). Based on our data, however, we cannot reject the hypothesis of an equal mutation rate in and out of exons (Fisher's exact test; higher‐confidence pool: *P* = 0.94; low confidence‐pool: *P* = 0.93), although this is admittedly based on very few mutations.

Differences in age and height among trees within a species (Whitham and Slobodchikoff 1981), as well as other environmental and genetic differences (e.g., Sharp and Agrawal 2012), are predicted to generate variation in somatic mutation rates among individuals and populations. The Sitka spruce trees in our study were similar in age and height, and they have almost certainly out‐lived the average generation time for this species, which may upwardly bias our mutation rate reported within a generation. Extrapolating our estimates to other trees may not be straightforward if mutations accumulate in a nonlinear manner with age or height (e.g., if older trees are more or less susceptible to mutagens such as fungal infections; Ranade et al. 2014) or if some environmental conditions result in higher mutation rates (e.g., at high elevations due to higher UV levels).

In contrast to the estimate within a generation, we infer a remarkably low annual somatic base substitution rate in Sitka spruce (Fig. 2B). This trend seems to be common among long‐lived organisms (e.g., trees and humans), although mutation rates have so far been reported for relatively few eukaryotes, and new estimates are accumulating rapidly. It is also in line with previous results that both height and genome size are negatively correlated with neutral substitution rates per year in both gymnosperms (de la Torre et al. 2017) and angiosperms (Bromham et al. 2015), with Sitka spruce being among the tallest trees and having a large genome (Birol et al. 2013). Meiosis aside, the per‐year mutation rate that we estimate in Sitka spruce is one‐hundredth of that reported for rice (*Oryza sativa*; Yang et al. 2015) and one‐third that of oak (Schmid‐Siegert et al. 2017), consistent with the much lower annual synonymous substitution rates reported for gymnosperms (de la Torre et al. 2017).

Our estimated mutation rate per year is, however, an order of magnitude lower than the neutral substitution rate inferred for spruce from molecular phylogenetics (8.03 × 10^−10^ per year; based on the fossil age of *Picea* and genetic data from *P. glauca*, *P. sitchensis*, and *P. abies*; de la Torre et al. 2017). Some of this discrepancy may be caused by rate variation among the species studied. Indeed, the three *Picea* species differ substantially in branch length from their common ancestor (Fig. 1 in de la Torre et al. 2017), with dS along the branch to the species studied here (*P. sitchensis*) being 40% shorter than the average. The remaining discrepancy may be explained by episodes of faster mutation in the history of the genus (e.g., because of shorter generation times or the transient appearance of a mutator allele), by the advanced age of the trees in this study, by purifying selection within meristems, or by our exclusion of the mutations that arise each generation during meiosis, as well as the mitoses involved in seed production and seedling growth.

That the somatic mutation rate is relatively high within a generation but low per year has various implications, according to the timescale of the evolutionary process in question. Branch lengths within a phylogenetic analysis are proportional to the substitution rate per unit time and so reflect the mutation rate per year rather than per generation. Similarly, tracking year‐to‐year variation in the environment may be challenging for long‐lived trees with a low annual mutation rate, limiting the ability of conifers to adapt to fast‐changing climates (Aitken et al. 2008) or to coevolve with insect herbivores (Whitham and Slobodchikoff 1981). For example, given the transcriptome size of Sitka spruce (182.2 Mb) and our estimated annual mutation rate, we predict only 0.027 genic mutations per year for a single diploid cell lineage in spruce, compared to an estimate of 5.2 in Arabidopsis (see Table S2; transcriptome size 33.2 Mb; The Arabidopsis Genome Initiative 2000).

Genetic load, by contrast, depends on the per‐generation rate of deleterious mutations (Morton et al. 1956), and conifers are known to have large genetic loads (Klekowski 1988). For example, the mean number of lethal equivalents (estimated as the total number of genes that contribute to increased mortality upon self‐pollination, weighted by their selective effect) has been estimated as 8.5 per diploid genome in loblolly pine (Franklin 1972) and 11.2 in Douglas‐fir (Sorensen 1969), compared to only 1.4 in humans (Bittles and Neel 1994). More recently, over 13% of all minor variants for SNPs in the exomes of two spruce species and their hybrids were bioinformatically predicted to be deleterious (Conte et al. 2017). Hence, the high rate of somatic mutation within a generation documented here would contribute substantially to the large genetic load observed in most conifers.

Somatic mutation is also of interest for adaptation, insofar as it produces new genetic variation. Using a simplified model that assumes the accumulation of somatic mutations is a function of shoot length alone (Supporting Information Note 3), we roughly estimated the total amount of genetic variation that somatic mutations might generate across all fertile branches of a tree within a generation. Approximating the number and length of first‐, second‐, and third‐order branches leading to seed or pollen cones over the life of an old‐growth Sitka spruce, we predict that on the order of 100,000 base substitutions could occur somewhere during development in total across all fertile branch lineages in these large trees. Indels, inversions, and structural changes would generate additional genetic variation. The vast majority of these mutations would occur late in development, however, affecting only a few cones, as the majority of the total branch length is amassed at the tips of branches.

Selection within or among meristems could act on such variation within an individual tree. Such selection would skew the distribution of fitness effects for heritable mutations by removing variants that are deleterious at the cell or branch level, and by improving the probability that beneficial mutations fix in meristematic cell lineages (Gaul 1964; Otto and Orive 1995). In particular, the mutation rates presented here could be underestimates if selection within or among meristems removed deleterious mutations. As noted above, we have no evidence that selection preferentially eliminated mutations within exons. Similarly, two of the three coding mutations observed were nonsynonymous, and they were not eliminated by selection (Table 2). Drawing firm conclusions about the efficacy of selection within individual trees based on these molecular signatures is not possible, however, with so few observed mutations.

The architecture and development of conifers must affect the efficacy of selection within the individual. The relatively large number of apical initials in conifers (20–30 in Douglas‐fir; Owens and Molder 1973) allows selection among cells within the meristem to be more efficient (Supporting Information Note 4; Fig. S1), although the highly structured nature of conifer apical meristems (Evert 2006) might limit the potential for cells with higher fitness to remain within the meristem. An even larger limitation on the potential for somatic selection in conifers like Sitka spruce is the strong apical dominance that hinders side branches from dominating a tree, unlike the multi‐stemmed structure common in many woody angiosperms. Side branches could nevertheless attain apical dominance when the dominant terminal leader is damaged or killed (e.g., by the spruce shoot tip weevil *Pissodes strobi*; Mitchell et al. 1974) or when apical dominance weakens within the crowns of old‐growth trees. Although rare “jackpot” mutations within the trunk or a prominent branch might proliferate and come to dominate a tree, most mutations will occur within secondary or tertiary branches that are unlikely to attain apical dominance no matter how much a mutation increases fitness.

Given the constraints on somatic selection in conifers, the importance of somatic mutation for adaptation may instead be dictated by its contribution to heritable genetic variation among the progeny of a tree. If most fertile branches contribute gametes to seeds that establish as seedlings—that is, despite the inefficiency of wind pollination, high rates of seed mortality, and seeds falling on unsuitable sites—then the progeny of a tree could contain a substantial pool of mutations produced over all of the branches of the parent (on the order of 100,000 base substitutions; above). Strong selection among seedlings (e.g., due to a mismatch with local environmental conditions for germination or a severe insect outbreak reducing viable seed production in nonresistant branches) would increase the probability that survivors bear very rare beneficial mutations. As a result of accessing a large pool of somatic mutations among seeds, conifers might display faster adaptive evolution than their long generation times would suggest.

The high somatic mutation rate of Sitka spruce within a generation must also generate much of the genetic variation that creates and maintains local adaptation. Because conifers often have large effective population sizes (Dodd and Silvertown 2000) and highly polygenic adaptive traits (White et al. 2007), a higher per‐generation mutation rate can strengthen adaptation (Yeaman 2015). Likewise, when adaptation requires a very rare beneficial mutation or sequence of mutations, somatic mutation improves the odds that it will appear somewhere within a population. High levels of gene flow via wind‐born pollen also facilitate the rapid spread of advantageous mutations among populations (Kremer et al. 2012).

A similarity between the spectrum of mutations observed from our study of Sitka spruce and in rice, oak, peach, and Arabidopsis is the preponderance of G/C to A/T transitions (Yang et al. 2015; Xie et al. 2016; Schmid‐Siegert et al. 2017; Exposito‐Alonso et al. 2018), which often result from errors replicating DNA that has been damaged by UV or by the deamination of methylated cytosines at CpG sites (Friedberg et al. 2006; Takuno et al. 2016). Because damage of this kind is thought to arise continuously over time, the results for all five species suggest that base substitutions in plants may originate primarily from misreplicating damaged DNA rather than mistakes during replication itself. Nonetheless, as modeled by Gao et al. (2016), substitution rates can still be proportional to the number of mitotic divisions (when repair rates are high and only the fraction unrepaired at the time of mitosis becomes mutation), even though the process generating damage is proportional to time.

Thus, unless repair is very slow, it is also of interest to estimate the mutation rate per cell division for comparison among plant taxa. Unfortunately, the number of cell divisions along a lineage leading from seedling to cone is unknown in conifers. At the lower bound, Burian et al. (2016) suggested six apical cell divisions per branching event based on studies of Arabidopsis and tomato, yielding about 18–24 cell divisions in the third‐ or fourth‐order branches sampled for this study. At the upper bound, if we extrapolate from maize (∼2 m tall; 50 mitotic divisions; Otto and Walbot 1990) to Sitka spruce (∼75 m tall) based on height, we expect ∼2000 mitotic divisions from one seed to the next. Bounding the number of cell divisions between 2000 and 20, we estimate between 1.4 × 10^–11^ and 1.4 × 10^–9^ mutations per cell division for the trees in our study. By comparison, the mutation rates per cell division in other taxa tend to be at the higher end of this range, varying from 4.4 × 10^–10^ to 9.8 × 10^–10^ in four single‐celled piko‐phytoplankton (Krasovec et al. 2017) and estimated as 2 × 10^–10^ in Arabidopsis (Ossowski et al. 2010).

These comparisons suggest that the number of mitotic divisions in spruce is an order of magnitude smaller than 2000. Indeed, if we were to assume that the mutation rate in plants is constant at 2 × 10^–10^ base substitutions per cell divisions (c.f. Arabidopsis), we would predict 135 mitoses within a generation for our samples from Sitka spruce. Consequently, cell divisions in a lineage of apical initials may happen years apart for ∼360‐year‐old trees. Our results are thus broadly consistent with the view that the apical meristems of tall conifers undergo fewer mitotic cell divisions than expected based on their height and, in so doing, bear fewer mutations per year.

We offer a direct genomic estimate of a somatic mutation rate in a conifer. The somatic base substitution rate is very high in old‐growth Sitka spruce within a generation but very low per year. Somatic mutations will considerably increase genetic load, which depends on the per‐generation deleterious mutation rate. The relevance of somatic mutations for adaptation to fast‐changing conditions is, however, limited by the long generation times and strong apical dominance of conifers, unless beneficial variants increase the proliferation of cells or branches within a tree and thereby their odds of persistence. Additionally, somatic mutations may facilitate adaptation when the few seeds that bear locally adapted mutations are substantially more likely to survive.

## CONFLICT OF INTEREST

The authors declare no conflict of interest.

Associate Editor: L. Bromham

## Supporting information

Supporting Information

Supporting Information

**Table S1**. Heights and branch lengths of the trees in this study.
**Figure S1**. The fixation probability of a mutation beneficial at the cell level divided by the fixation probability of a neutral mutation $P_{sel}/P_{neutral}$ versus the mutation's selective advantage at the cell level (*s*).η is the effective number of replicating apical initial cells (see Supporting Information Note 4).
**Table S2**. Data and references for Figure 2.
**Table S3**. Values for the calculation of the heterozygosity correction used for the calculation of the reported mutation rates (see Supporting Information Note 2).
**Table S4**. Alternative values for the calculation of the heterozygosity correction (see Supporting Information Note 2).
