# Supplementary figures and images for: Somatic mutations substantially increase the per‐generation mutation rate in the conifer Picea sitchensis
Source: Evol Lett. 2019 Jun 10;3(4):348–58. doi: 10.1002/evl3.121 (PMC6675141; doi:10.1002/evl3.121)

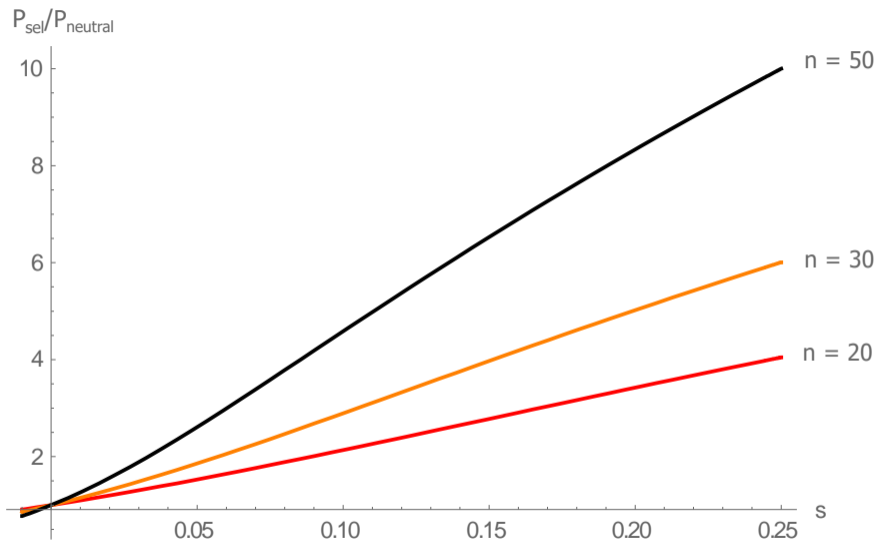

Supplement: Supplementary file 1 — Supporting Information [file EVL3-3-348-s001.pdf]
