# Supplementary material for: Somatic mutations substantially increase the per‐generation mutation rate in the conifer Picea sitchensis
Source: Evol Lett. 2019 Jun 10;3(4):348–58. doi: 10.1002/evl3.121 (PMC6675141; doi:10.1002/evl3.121)
Supplement: Supplementary file 3 — Table S1. Heights and branch lengths of the trees in this study. Figure S1. The fixation probability of a mutation beneficial at the cell level divided by the fixation probability of a neutral mutation Psel/Pneutral versus the mutation's selective advantage at the cell level (s).η is the effective number of replicating apical initial cells (see Supporting Information Note 4). Table S2. Data and references for Figure 2. Table S3. Values for the calculation of the heterozygosity correction used for the calculation of the reported mutation rates (see Supporting Information Note 2). Table S4. Alternative values for the calculation of the heterozygosity correction (see Supporting Information Note 2). [file EVL3-3-348-s003.pdf]

Supplementary Material

Note 1: Genetic heterogeneity of bark samples

Trees grow in diameter when new wood cells are added to the ends of long radial cell files, extending horizontally from the pith towards the bark (see Fig. 12.1 *A* and Fig 12.6 *B* in Esau's Plant Anatomy[1] to visualize this growth pattern). These cell files are produced by periclinal divisions of stem cells in the vascular cambium that also divide to produce secondary phloem (inner bark) to the outside of the vascular cambium. These meristematic cambial cells must periodically divide anticlinally to accommodate increases in circumference, with each doubling of the diameter requiring each cambial cell to divide anticlinally once, on average, doubling the number of cambial cells. This pattern of growth causes the cell lineages to exhibit a star-like genealogical tree[2], with coalescent events for the cells in a ring of bark clustered near the most recent common ancestor, representing cells near the pith of the tree where the most diameter doublings occurred.

Our bark samples in this study are disks of cambial and phloem tissue with a diameter of approximately 2 cm taken from the outer circumference of the tree. The vast majority of cambial cells are fusiform initials, which divide to produce mother cells that produce tracheids in wood and longitudinal cells in phloem (sieve cells, parenchyma cells and fibers). Fusiform initials average the same size as tracheids, approximately 0.03 mm wide in Sitka spruce[3]. Across the diameter of this disk, there would be approximately 667 fusiform initial cells in the cambium. These cells are therefore separated from their most recent common ancestor by ~9 doublings of the diameter ($2^{9.38} = 667$). For a tree with a radius ~1m, this implies that the most recent common

---

[1] Evert RF (2006) *Esau's Plant Anatomy*. 324-328
[2] Slatkin M, Hudson RR (1991). Genetics 129:555-562
[3]e.g. Mitchell MD, Denne MP (1997). *Forestry: An International Journal of Forest Research 70:47-60*

ancestor of these cells is ~1.5 mm from the pith of the tree on average. Thus, each bark sample contains distantly related cell lineages that diverged very early in the life of the tree and are separated by nearly as many cell divisions as any pair of cells on opposite sides of the trunk (even more so because cell files at different heights within a bark sample are more distantly related than cell files in the same row, and as fusiform initials are 1-3 mm long with overlapping rows, our 2 cm sample would include approximately 20 rows). This leads us to predict that genetic variation within a bark sample should be nearly as great as genetic variation between a pair of bark samples. In particular, the called genotype of a bark sample is a majority genotype that reflects the most recent common ancestor of the sample, a cambial cell that produced wood near the pith. This minimizes the difference between the two bark samples. It is thus impractical to compare the genotypes of two bark samples to obtain an estimate of the somatic mutation rate across the trunk of a tree without much greater depth of coverage allowing low-frequency somatic mutations present in just a few cells to be detected.

Note 2: The size of the search space

The heterozygosity correction to the size of the search space is derived from the observed average decrease in heterozygosity caused by the genotype-level filters applied to variable sites. If these filters discard a proportion p of homozygous sample genotypes and a proportion q of heterozygous sample genotypes (where a sample genotype is specific to one of the four samples from a tree at a genomic site; in our study, $q > p$), and if all four sample genotypes for any tree genotype must pass all filters if the tree genotype itself is to pass filters, then $(1-p)^4$ of four-sample homozygous tree genotypes and $(1-q)^4$ of four-sample heterozygous tree genotypes would pass filters. Both quantities can be directly estimated from the numbers of heterozygous and homozygous consensus tree genotypes (i.e., all four samples agree) present after the site-

level filters (n_{het} and n_{hom}) and after both the site- and genotype-level filters (n'_{het} and n'_{hom}), allowing p and q to be estimated (note that n_{het} and n_{hom} do not sum to V, which is calculated after the all sites filters; see Table 1) . Because mutations consist of two heterozygous and two homozygous genotypes, a fraction $y = (1-p)^2(1-q)^2$ of the mutations would pass filters, where:

$$y = \sqrt{\frac{n'_{het} * n'_{hom}}{n_{het} * n_{hom}}}$$

The number of higher-confidence variable tree genotypes we would expect to have obtained if all tree genotypes passed filters at the rate that mutations do (i.e., at a constant rate of $(1-p)^2(1-q)^2$) is then equal to:

$$\sigma = y * (n_{het} + n_{hom})$$

We can thus correct the actual number of filtered tree genotypes, $n'_{het} + n'_{hom}$, by multiplying it by a correction factor, h, that would give σ as the expected total number of filtered tree genotypes, $h(n'_{het} + n'_{hom}) = \sigma$. Rearranging, this correction factor is:

$$h = \frac{\sigma}{n'_{het} + n'_{hom}} = \frac{(n_{het} + n_{hom}) * \sqrt{n'_{het} * n'_{hom}}}{(n'_{het} + n'_{hom}) * \sqrt{n_{het} * n_{hom}}}$$

For the final calculation of the search space, we calculated h (and y) separately for rare and frequent sites. In particular, we restricted n_{het} and n_{hom} (and n'_{het} and n'_{hom}) to tree genotypes contained in either rare (MAF ≤ 0.05) or frequent (MAF > 0.05) sites to obtain $\sigma = \sigma_{rare}$ and $\sigma = \sigma_{freq}$, as seen in the Methods. The values of n, h, and σ are reported in Table S3. Note in h>1 for rare sites and h<1 for frequent sites in Table S3, implying that heterozygotes are affected differently by the rare and frequent filters (although the effect is reversed in the alternative

calculation presented below and in Table S4). To obtain the average heterozygosity correction for the higher-confidence pool reported in the text, we divided $\sigma_{rare} + \sigma_{freq}$ by the sum of $n'_{het} + n'_{hom}$ across rare and frequent sites (yielding 0.93) and repeated this procedure for the lower-confidence pool (yielding 0.99).

Note that in calculating the search space, we used *I* and *V* after the all sites filters (Table 1) rather than from the starting numbers of invariant and variable sites. We did this because the all sites filters eliminate many of the alignment and sequencing errors that cause sites to falsely appear to be variable. Our goal in this note was to explore the impact of the genotype-level filters on the number of retained sites (which could remove true variable sites), so that we could reduce the search space accordingly. If we repeat the search space calculations using *I* and *V* before any filters are applied (other than requiring data for all four samples and removing indels and multiallelic sites; Table S4), we obtain smaller estimated search spaces of 36.6 Mb (high-confidence) and 68.8 Mb (low-confidence), increasing the mutation rate estimates by roughly 20-50%. These estimates are less reliable, however, as *V* is expected to include more alignment and sequencing errors than *I* does. Thus, we report all results using *I* and *V* after the all sites filters to eliminate many of these false variable sites.

Note 3: The total mutation rate

In this section, we estimate the total number of heritable mutations expected across all branches of a tree, given the inferred mutation rate of 2.7 x 10^{-8} per base pair observed between the base and the upper crown of tall Sitka spruce. We assume that the mutation rate is homogeneous over time and the age of the tree, that it scales approximately with branch length, and that selection is absent. We focus only on mutations within meristems in branches that could give rise to seed or pollen cones. In Sitka spruce, seed cones are restricted to the upper portion of

the crown, but we also count mutations that arise in branches lower in the tree (excluding the lowest portion of the stem) as they may bear pollen cones and would have been in the upper portion of the crown at an earlier point in the life cycle. We disregard the additional mitotic divisions required for seed or pollen production.

Based on visual inspections of typical spruce tree shapes, we build a rough model of the total length of branches that could give rise to a heritable mutation as follows. Sitka spruce develop from a main stem (zero-order branch) of measured length $L0 = 76$ m for the trees in this study. We assume that first-order branches emerge from the main stem at a density of approximately $N1 = 5$ per meter and are roughly of length $L1 = 3$ m (we ignore all branches in the first 15 m of the main stem as these are unlikely to be reproductive). We assume also that second-order branches arise from these first-order branches at roughly $N2 = 10$ per meter and are roughly a length of $L2 = 0.5$ m. These second-order branches themselves can branch multiple times before giving rise to cones; this augments the total branch length borne by second-order branches by a factor of roughly $2 \leq f \leq 5$. Note that this model counts any one branch only once (e.g., the trunk), as mutations that occur along it would be shared by at least some of the branch tips.

Across a tree, then, the total shoot length across which mutations could occur is:

$$L = L0 + (L0 - 15) * (N1 * L1) + (L0 - 15) * (N1 * L1) * (N2 * L2 * f)$$

where the first term is the full length of the main stem, the second term is the length of all first-order branches higher than 15 m, and the third term is the length of all branches of second order or higher (again, above 15 m on the trunk). Evaluating this expression, we estimate that L is between 10,207 m and 24,022 m. If we divide by the total average length of the main stem and

sampled branch between the bark and foliage samples collected in this study ($LS = 74$ m), we obtain

$$137.0 \leq \frac{L}{LS} \leq 322.5$$

That is, given our assumption that the mutation rate scales with branch length, we expect that the mutation rate across all branches of a tree is approximately two orders of magnitude higher than the mutation rate to a single branch tip of a Sitka spruce tree (2.7 x 10^{-8} per base pair). Given that the latter corresponds to 1134 mutations across the entire diploid genome of Sitka spruce, we predict that on the order of 100,000 mutations occur across all fertile branches of a tree, mostly in small mutant sectors near branch tips.

Note 4: Selection among cells within meristems

Consider a single apical meristem bearing a new mutation. If the mutation is fixed within the meristem, descendent branches and cones will carry the mutation and allow it to be inherited. If it is lost, it will produce a limited mutant sector that may not bear cones. The exact nature of cell replication within the apical meristem of conifers is unknown, but we approximate it as a Moran process among n cells. In this process, the number of cells in the meristem is kept constant by coupling the replication of a random cell with the "death" of another random cell (here "death" describes the loss of a cell from the meristem, rather than cell death *per se*). n represents the effective number of cells that can contribute to replication within the apical meristem and has been estimated in Douglas-fir to be $20-30$ (Owens and Molder 1973).

If the mutation increases the growth rate of a cell, it is more likely to fix within the meristem in which it arose. Assuming that the relative chance of a cell being chosen for the next birth is increased by a factor $w = 1 + s$ relative to non-mutant cells, the fixation probability of

the mutation is $P_{sel} = \frac{w^{-1}-1}{w^{-n}-1}$ (e.g., Otto and Day 2007, eq. 14.58b). Neutral mutations fix with a probability of $P_{neutral} = \frac{1}{n}$, so selection within a meristem alters the fixation probability of mutations by a factor $P_{sel}/P_{neutral} = n * \frac{w^{-1}-1}{w^{-n}-1}$ (Fig. S1). Thus, selection increases the representation of beneficial mutations by a factor slightly less than $n * s$. Although this factor is not large when selection is weak and apical meristems are small, it can considerably increase the probability of fixation with strong selection (e.g., by a factor of three for $s = 0.1$ and a factor of ten for $s = 0.5$ with $n = 30$ cells within an apical meristem).

Supplementary Tables and Figures

Table S1 Heights and branch lengths of the trees in this study (*in separate Excel file).*

Fig. S1 The fixation probability of a mutation beneficial at the cell level divided by the fixation probability of a neutral mutation ($P_{sel}/P_{neutral}$) versus the mutation's selective advantage at the cell level (s). n is the effective number of replicating apical initial cells *(in separate PDF file).*

Table S2 Data and references for Fig. 2.

Species	μ per gen.	μ per year	Estimated age (years)	Notes
Heliconius Melpomene	2.90 x 10^{-9} [4]	1.16 x 10^{-8}	0.25 [5]	Assumes 6-month lifespan and continual reproduction
Mus musculus	5.40 x 10^{-9} [6]	8.64 x 10^{-9}	0.25 [7], 1.0 [8]	Mean generation time
Drosophila melanogaster	5.49 x 10^{-9} [9]	3.14 x 10^{-8}	0.1 [10], 0.25 [11]	Mean generation time
Apis mellifera	6.80 x 10^{-9} [12]	4.53 x 10^{-9}	1.5 [13]	Assumes 3-year lifespan and yearly swarming
Arabidopsis thaliana	7.00 x 10^{-9} [14]	7.78 x 10^{-8}	0.5 [15], 1.3 [16]	Mean generation time
Oryza sativa	7.10 x 10^{-9} [9]	7.89 x 10^{-9}	0.9 [17]	Harvest frequency
Prunus mira	9.48 x 10^{-9} [18]	4.74 x 10^{-11}	200 [15]	
Pan troglodytes	1.20 x 10^{-8} [19]	5.00 x 10^{-10}	24 [16]	
Homo sapiens	1.20 x 10^{-8} [20]	4.04 x 10^{-10}	29.7 [17]	
Picea sitchensis	2.65 x 10^{-8} [21]	7.36 x 10^{-11}, 8.03 x 10^{-10} [22]	360 [23]	Midpoint of the age range (220-500 years) for similar old-growth trees in the same stand, obtained from existing dendrochronology data. Because of the diameter of the trees in this study and their importance for Carmanah Walbran Provincial Park, we did not take increment cores from the trees themselves.
Quercus robur	4.25 x 10^{-8} [24]	1.82 x 10^{-10}	234 [21]	

[4] Keightley PD, et al. (2015). Mol. Biol. Evol. 32:239-234
[5] Boggs 1979, cited in Naisbit RE, Jiggins CD, Mallet J (2001). Proc. R. Soc. Lond. B 268:1849-1854
[6] Uchimura A, et al. (2015). Genome Res 25:1125-1134
[7] Castiglia R, Capanna E (1999). Heredity 83:319-326
[8] Geraldes A, et al. (2008). Mol Ecol 17:5349-5363
[9] Schrider DR, et al. (2013). Genetics 194:937-954
[10] Nei M, Maruyama T, Chakraborty R (1975). Evolution 29:1-10
[11] Keightley PD (1994). Genetics 138:1315-1322
[12] Yang S, et al. (2015). Nature 523:463–467
[13] Seeley TD (1978). Oecologica
[14] Ossowski S, et al. (2010). Science 327: 92–94
[15] Thompson L (1994). J. Ecol. 82: 63-68
[16] Falahati-Anbaran M, Lundemo S, Stenoien HK(2014). New Phytologist 202: 1043-1054
[17] Davis KF, Gephart JA, Gunda T (2016). Ambio 45:302-312
[18] Xie Z, et al. (2016). Proc R Soc Biol Sci 283: 1–9
[19] Venn O, et al. (2014) Science 344: 1272-1275
[20] Kong A, et al. (2012). Nature 488:471-475
[21] This study
[22] De La Torre A, Li Z, Van de Peer Y, Ingvarrsson P (2017). Mol Biol Evol 34: 1363–1377 (based on a phylogenetic analysis using three species of *Picea*)
[23] Little PJ, Richardson JS, Alila Y (2013). Geomorphology 202:86–100.
[24] Schmid-Siegert E, et al. (2017). Nat Plants 3(12): 926–929.

Table S3 Values for the calculation of the heterozygosity correction (Note 2) used for the calculation of the reported mutation rates.

	Higher-confidence		Lower-confidence	
	**Rare**	**Frequent**	**Rare**	**Frequent**
n_{het}	130971	713199	146156	761229
n_{hom}	3801449	2589523	3930628	2715380
n'_{het}	56270	69207	102524	182365
n'_{hom}	1457703	708318	2228352	1248811
$\boldsymbol{h}$	1.054	0.692	1.103	0.806
$\boldsymbol{\sigma}$	1596139	538079	2570888	1153990

Table S4 Alternative values for the calculation of the heterozygosity correction (Note 2). Note that n_{het} and n_{hom} are measured before the filtering (i.e., before sites are split into rare and frequent pools) and hence refer to the same total pool of variable sites, whereas n'_{het} and n'_{hom} involve different subsets of these sites for rare and frequent minor alleles.

	Higher-confidence		Lower-confidence	
	**Rare**	**Frequent**	**Rare**	**Frequent**
n_{het}	1320612	1320612	1320612	1320612
n_{hom}	10720380	10720380	10720380	10720380
n'_{het}	56270	69207	102524	182365
n'_{hom}	1457703	708318	2228352	1248811
$\boldsymbol{h}$	0.605	0.911	0.656	1.067
$\boldsymbol{\sigma}$	916521	708531	1529587	1527175
